# Impact of treatment with direct-acting antivirals on anxiety and depression in chronic hepatitis C

**DOI:** 10.1371/journal.pone.0208112

**Published:** 2018-12-19

**Authors:** Marta Gallach, Mercedes Vergara, Joao Pedro da Costa, Mireia Miquel, Meritxell Casas, Jordi Sanchez-Delgado, Blai Dalmau, Núria Rudi, Isabel Parra, Teresa Monllor, Meritxell Sanchez-Lloansí, Angelina Dosal, Oliver Valero, Xavier Calvet

**Affiliations:** 1 Hepatology unit, Digestive Disease Department, Parc Taulí Hospital Universitari, Institutd’Investigació i Innovació Parc Taulí I3PT, Universitat Autònoma de Barcelona; Sabadell, Spain; 2 CIBERehd, Instituto Carlos III, Madrid, Spain; 3 Pharmacy Department, Parc Taulí Hospital Universitari, Institutd’Investigació i Innovació Parc Taulí I3PT, Universitat Autònoma de Barcelona, Sabadell, Spain; 4 Mental Health Department, Parc Taulí Hospital Universitari, Institutd’Investigació i Innovació Parc Taulí I3PT, Universitat Autònoma de Barcelona, Sabadell, Spain; 5 Nursing, Hepatology Day Hospital, ParcTaulí Hospital Universitari, Institutd’Investigació i Innovació Parc Taulí I3PT, Universitat Autònoma de Barcelona, Sabadell, Spain; 6 Statistical services center, Universitat Autònoma de Barcelona, Barcelona, Spain; Kaohsiung Medical University, TAIWAN

## Abstract

**Background and aim:**

Treatment of hepatitis C with direct-acting antiviral agents (DAA) has few side effects. Although pivotal studies suggested that DAA were safe in patients with psychiatric diseases who could not be treated with previous antiviral therapies, their effects on anxiety and depression have not yet been analysed in clinical practice. The aim of our study was to analyse anxiety and depression in the setting of DAA treatment in a clinical practice series.

**Methods:**

All patients starting DAA treatment between November 1, 2014 and October 31, 2015 were eligible. Patients completed the Hospital Anxiety and Depression scale at different times during treatment. The results were plotted on line graphs and evaluated using a linear regression model with repeated measures.

**Results:**

One hundred and forty-five patients were included (11% with major psychiatric disorders; 32% on psychiatric treatment). Sustained virologic response (SVR) was achieved in 97.3% of cases. Anxiety and depression measures did not differ between time points. No differences between patients on psychiatric treatment or with advanced fibrosis or cirrhosis were found at any time point analysed.

**Conclusion:**

DAA treatment had no impact on anxiety or depression during or after chronic hepatitis C infection treatment, even in high-risk patients with major psychiatric disorders.

## Introduction

Hepatitis C is a major cause of chronic liver disease worldwide,[1–5] with an estimated 71 million people infected in 2015[6] and approximately 1.75 million new infections each year.(3) Chronic hepatitis C virus (HCV) infection is a leading cause of cirrhosis in the United States and Europe, and is associated with increased liver-related and overall mortality[4, 7, 8]. Antiviral treatment has the potential to improve long-term outcomes for patients with cirrhosis, and viral clearance is associated with histological improvement, decreased risk of hepatocellular carcinoma, reduced incidence of decompensation, and lower liver-related and non-liver-related mortality[9–11]. Van der Mer et al[12]. found an association between sustained virologic response (SVR) and a decrease in all-cause mortality among patients with chronic HCV infection and advanced hepatic fibrosis. In addition to physical symptoms, HCV-infected patients suffer fatigue, depression and anxiety that can impair health-related quality of life, lower work productivity and worsen patient-reported outcomes.[1]

Direct-acting antiviral agents (DAA) enable higher cure rates with fewer side effects and shorter treatment[1, 5]. DAAs were associated with reductions of over 30% in waiting lists for liver transplantation for HCV cases complicated by decompensated cirrhosis[13] Replacing pegylated interferon and ribavirin with DAAs has substantially improved health-related quality of life during treatment,[14, 15] and may result in better patient experience and higher treatment adherence.[3, 14] DAA treatment is associated with substantial improvements in patient-reported outcomes (PRO) during therapy that continues long after a sustained viral response (SVR) is achieved.[1, 16]

Patients with severe comorbid mental illness have historically been considered at high risk for HCV treatment, mainly because of the significant neuropsychiatric adverse effects associated with pegylated interferon.[2, 3, 15] As interferon-free regimens do not appear to be related to neuropsychiatric adverse effects, they can be administered to patients with severe mental illness and/or substance abuse.[2, 3, 15] However, pre-existing psychiatric issues may affect tolerability of therapy, and central nervous system drugs (mainly anti-psychotics and anti-depressants) can also interact with DAA therapy.[17–19] Nevertheless, these potential interactions can be well controlled, because the most common antidepressants or benzodiazepines administered have few interactions with DAA. Moreover, the different combinations of DAA allow one to be administered that does not interact with the patient’s psychiatric medication. Yek et al.[20] recently published their experience in clinical practice, where 38% of their patients presented with mental health disorders. The SVR in these patients was 89%, similar to that of the patients without mental illness.

Patients selected for inclusion in DAA clinical trials are often not representative of the complex variability seen in clinical practice, where patients are frequently older and/or sicker than those in clinical trials.[1, 5] In addition, clinical trials generally do not focus on measuring anxiety or depression in patients on DAA treatment, and mental disorders are generally an exclusion criterion. Real world evidence on these points is scant. Sundberg et al.[15] published a prospective study that was the first to specifically examine psychiatric symptoms during DAA treatment in real-world patients with substantial psychiatric morbidity (including a history of substance abuse). In this small study (*n* = 17), DAA treatment in patients with significant psychiatric comorbidity did not increase depressive symptoms or affect sleep quality. Treatment adherence was very high (> 95%), as was the treatment response (88%).

The primary aim of this study was to evaluate the change in anxiety and depression scores over the course of DAA treatment and after its completion in patients with chronic HCV infection in clinical practice. Secondary aims were to evaluate the tolerability, adherence and response in patients with severe mental disorders and advanced liver disease.

## Methods

### Patients

All HCV patients who initiated DAA treatment in our hepatology outpatient unit between November 1, 2014 and October 31, 2015 were included in the study. A signed informed consent was mandatory to be eligible. Exclusion criteria included: coinfection with HIV or hepatitis B virus (to avoid the possible effects of coinfection on anxiety and depression before and during DAA treatment), and inability to understand and complete the questionnaires.

The study was performed in the hepatology outpatient unit of Hospital Parc Taulí in Sabadell, a city 15 km from Barcelona.The hospital covers a population of 400 000 inhabitants.

### Study design

This was a prospective observational study, conducted following approval by the hospital ethics committee (Comite Ètic d'Investigació Clínica de la Corporació Sanitària Universitària Parc Taulí, approval number 2010/612). The DAA treatment administered to the patients was determined by the physician according to guidelines, and administered as per standard clinical practice. Routine management of HCV infection in our hepatology unit at the time of the study involved a multidisciplinary team including pharmacists, psychiatrists, nurses, and hepatologists. The pharmacists dispensed medication and evaluated treatment adherence and the potential interactions of DAAs with other drugs; the nurses were responsible for patient education and follow-up, and reinforced the information given by the hepatologist and pharmacist; the hepatologists evaluated the patients, prescribed treatment according to both patient and infection characteristics, and coordinated care during and after treatment, thus overseeing the entire process; and the psychiatrists prospectively evaluated patients prior to DAA treatment and during follow-up when necessary.

### Evaluation of anxiety and depression

Before starting DAA treatment, all patients were evaluated in our hepatology outpatient unit using a standardized form (S1 Annex) to determine their social and psychiatric status, and whether they were able to sign the informed consent form. To measure their anxiety and depression status, patients completed the validated Spanish version of the Hospital Anxiety and Depression Scale (HADS)[21–23]. This is a self-administered 14-item questionnaire [21, 22], divided into two 7-item scales designed to screen and rate depression (HADS-D) and anxiety (HADS-A) in medical patients.[21, 22, 24]. Each item is scored from 0 to 3 points, with a higher score indicating more depression or anxiety. A score greater than 7 in one of the scales (depression or anxiety) indicates susceptibility to depression or anxiety. HADS was administered at the start of treatment (baseline) by the hepatologist. If patients scored more than 7 in depression or anxiety, they were evaluated by the psychiatrist to assess the need for psychiatric treatment, re-evaluate current psychiatric treatment or to consider special psychiatric follow-up during DAA treatment. HADS was administered by pharmacists in week 4 of treatment, week 12 of treatment, and/or at the end of treatment, before refilling patients’ prescriptions for DAA. The pharmacist also administered HADS during a follow-up visit 12 weeks after the end of treatment. Patients did not know the results of treatment before completing the questionnaires. Patients who did not complete at least three out of five HADS assessments were excluded from the final analyses of anxiety and depression. The questionnaires were completed voluntarily by the patients. Patients with psychiatric disorders were defined according to the WHO classification for major psychiatric disorders, which was the system generally used by the psychiatrist [25]. Those with at least one diagnosis in the ICD-10 classification of mental and behavioural disorders [25] were classified as having a severe mental disorder.

#### Measurable outcomes

Demographic data, alcohol consumption and medical history (including mental illnesses and psychiatric treatment) before and during DAA treatment, HCV characteristics (genotype and viral load), fibrosis stage, treatment adherence and response to DAA treatment were recorded. To evaluate the response to treatment, viral load (HCV-RNA) was measured 12 weeks after the end of treatment. which has a lower limit of detection of < 15 IU/ml. Sustained viral response (SVR) was defined as undetectable HCV RNA 12 weeks after the end of therapy [26]. Most patients were evaluated by elastography to evaluate the fibrosis stage. Values of ≥9.6 and ≥14 were considered as advanced fibrosis and cirrhosis, respectively. To evaluate treatment adherence, all patients answered the Spanish version of the Morisky-Green test, a validated questionnaire translated and validated into the Spanish language [27, 28].This test has the advantage of providing information on the causes of non-adherence. Affirmative answers were scored 0 points and negative answers 1 point. Patients who answered yes to all questions (total score 0 points) were considered adherent.

The primary endpoint was to assess the change in anxiety and depression scores during DAA treatment and until 12 weeks after the end of treatment. The secondary endpoint was the SVR rate 12 weeks after the end of treatment. We also analysed prognostic factors for anxiety or depression during treatment.

### Antiviral treatment and dosage

Individual treatment was based on European and Spanish guidelines.[29, 30] The length of treatment ranged from 8–24 weeks depending on viral genotype, previous treatment and fibrosis stage. Patients were visited by the hepatologist at baseline, week 4, week 12,week 24 or at the end of treatment, and 12 weeks after the end of treatment. During the visits, the hepatologist evaluated symptoms, reinforced adherence and evaluated blood tests or other additional examinations such as abdominal ultrasound.

### Statistical analysis

Descriptive statistics included frequencies and percentages for categorical variables and means and standard deviations for quantitative variables. Bivariate analyses were used to compare baseline characteristics between patients who completed at least three HADS questionnaires and those who completed two or less. The Chi-square test was used for categorical variables, and t-tests/Wilcoxon tests for quantitative variables.

A regression model with repeated measures was performed to analyse HADS-A and HADS-D over time.[31]Two linear regression mixed models were used to evaluate differences in HADS-A and HADS-D over time. Time was considered as a fixed effect and patient as a random effect to account for repeated measures. Additional linear mixed models were used to analyse the interactions between time and the variables treatment duration, psychiatric treatment, and fibrosis stage. Data was graphically represented using boxplots. Statistical significance was set at 0.05 for all tests. The analysis was performed with software SAS v9.4 (SAS Institute Inc. Cary, NC, USA).

## Results

During the one-year inclusion period, 230 patients started DAA treatment; of these, 85 were excluded from the analyses because they did not complete at least three HADS questionnaires. Thus, 145 patients were eventually analysed. Reasons for not completing questionnaires were diverse and not recorded in our database, but included leaving the questionnaire at home and lack of interest since the treatment was well-tolerated.

### Characteristics of included and excluded patients

**Fig 1:**
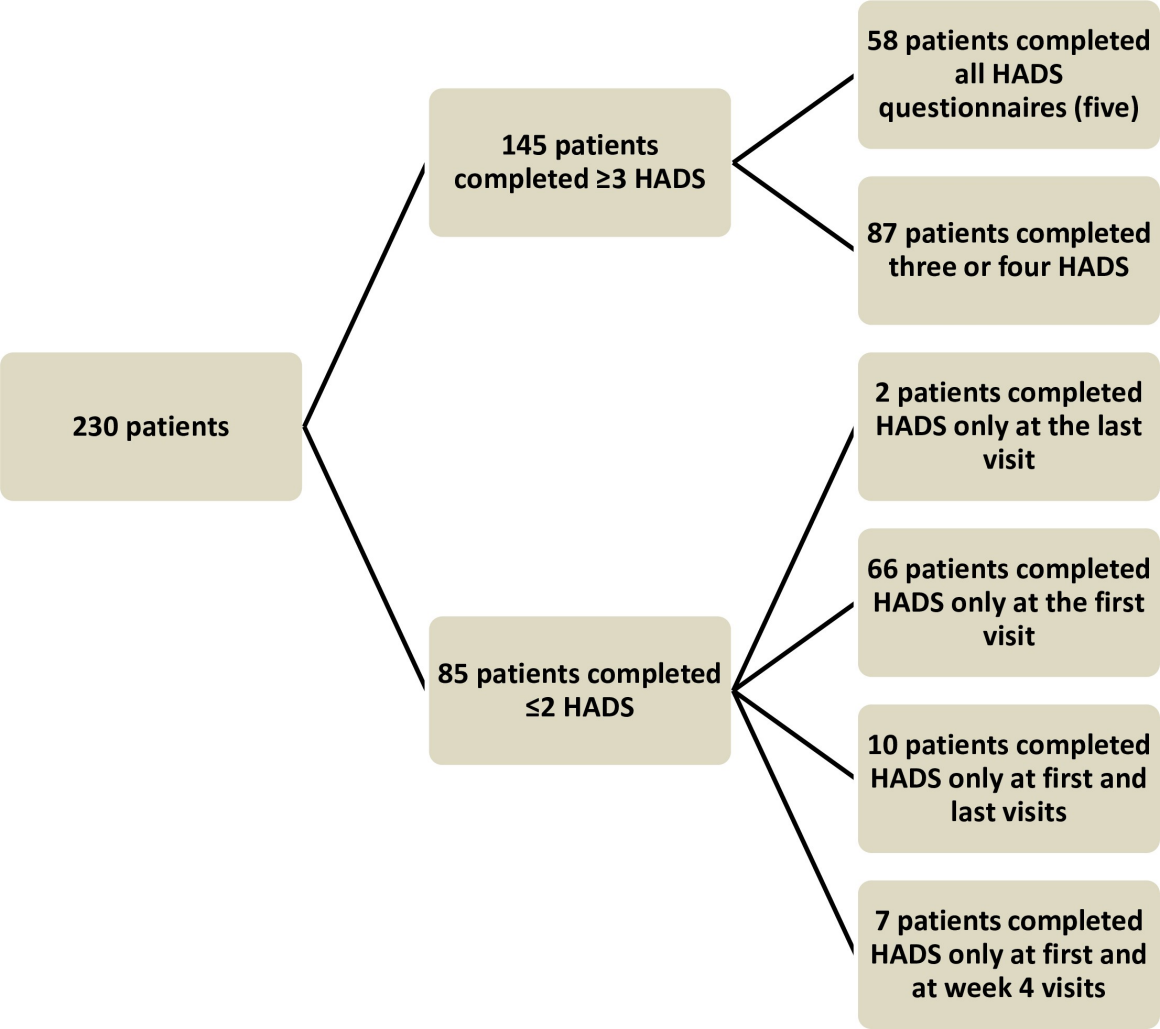
Flow chart showing the number of surveys completed by patients.

Fig 1 shows the breakdown of the number of surveys that patients completed. To check for inclusion bias, we analysed the differences between the 145 patients who completed at least three of the four HADS questionnaires and the 66 excluded patients who completed the initial survey only. There were no significant differences in age (58.6 ± 10.5 *vs*. 61.5 ± 11.1, *P* = 0.08), genotypes (*P* = 0.16), values for the controlled attenuation parameter on elastography (17.6 ± 11.5 *vs*. 21.3 ± 15, *P* = 0.101), or receiving previous treatment (26.4% *vs*. 73.6%, *P* = 0.1) in the group of excluded patients. Moreover, no differences were detected in the number of patients with previous history of mental illness (27 excluded patients *vs*. 37 included, p = ns). In addition, the baseline scores for depression (4.55±4.13 *vs*. 4.07±3.82) and anxiety (6.20±4.15 *vs*. 6.25±4.81) were similar for included and excluded patients. The only significant difference between included and excluded patients was found in those patients born outside Spain, who were less likely to complete the questionnaires, probably due to difficulties with language.

### Demographic and clinical characteristics of included patients

Of the 145 patients included (mean age, 61.5±11.1 years); 81 (55.9%) were men; 94 (64.5%) were married; 108 (85.7%) had children; 142 (98.4%) were born in Spain; and 84 (57.9%) were retired. Genotype 1b was the most frequent; it was present in 99 (68.3%) patients. Elastography indicated cirrhosis in 85 (62%) patients (Table 1). Mean Child Pugh index score was 5.4±0.8 points and mean MELD score was 9.5±2.7 points.

**Table 1:** Demographic, clinical and virologic patient characteristics.

Variable	
Age in years, (mean ± *SD)	62 ± 11.11
Men (n = 145)	81 (55.9%)
Genotype: (n = 145)	
1a	17 (11.7%)
1b	99 (68.3%)
2	5 (3.4%)
3	12 (8.3%)
4	10 (6.9%)
¼	1 (0.7%)
Undetermined	1 (0.4%)
Transient elastography (mean ± *SD)	16.9 ± 15.07 kPA
F4 (> 14 KPa) (n = 137)	85 (62%)
Endoscopy: (n = 104)	97 (66.9%)
Esophageal varices	53 (51%)
Portal gastropathy	18 (17.5%)
Previous antiviral treatment **(pegylated interferon and ribavirin)** (n = 145)	81 (73,6%)
Null responder	37 (45.7%)
Relapsed	23 (28.4%)
Treatment discontinued due to intolerance	16 (19.7%)
Partial response	5 (6.2%)
**Mental diseases:** (n = 145)	46 (31.7%)
** Depression**	19(51.4%)
** Anxiety**	12 (32.4%)
** Psychotic**	7 (18.9%)
** Drug abuse**	6 16.2%)
** Personality disorder**	1 (2.7%)
** Current severe mental disorder**	16(11%)
**Psychiatric medication** (n = 145)	42 (29.4%)
Social characteristics:	
Married / living with partner (n = 121)	80 (64.5%)
Children, mean ± *SD (n = 126)	2 ± 1.34
Born in Spain (n = 126)	124 (98.4%)
Completed primary school (n = 125) High school and University (n = 125)	74 (59.2%) 34 (27.2%)
Employment status: (n = 126)	
Retired	73 (57.9%)
Active	34 (27%)
Unemployed	12 (9.5%)
Homemaker	7 (5.6%)

*SD, Standard deviation

### DAA treatment administered

**Fig 2:**
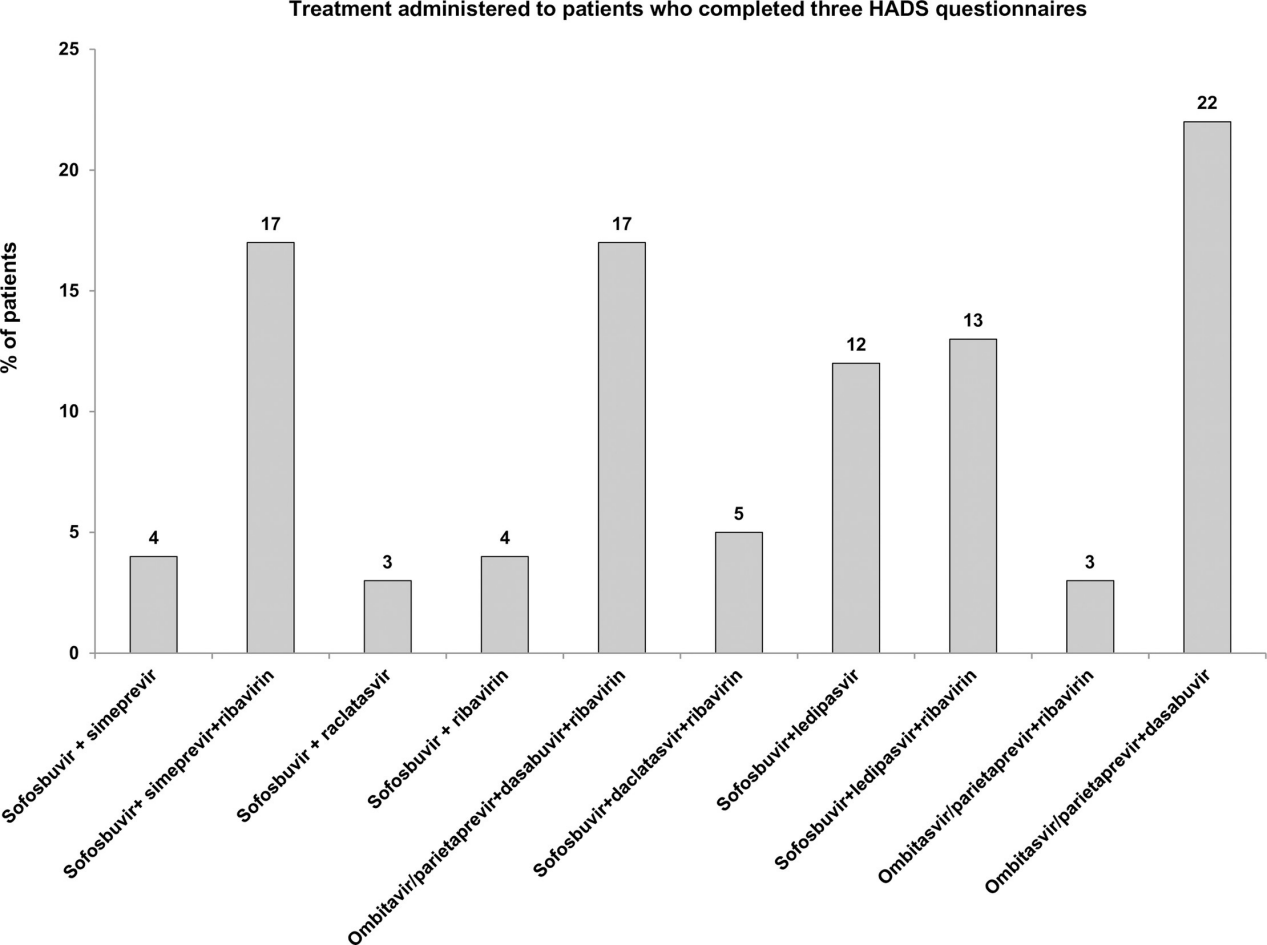
Treatment administered to patients who completed three HADS questionnaires. Percentage of patients are specified for every treatment. **HADS**, Hospital Anxiety and Depression Scale score.

Data on treatment are shown in Fig 2. A total of 105 (72.4%) patients received 12 weeks of treatment and 40 patients (27.6%) received 24 weeks. No patient received treatment for 8 weeks. Eighty-four (58%) patients received ribavirin adjusted according to weight (ribavirin 1000mg per day in two separate doses if less than 75 kg or 1200mg if equal to or greater than 75 kg). Ribavirin dose was reduced to 600 mg per day in the case of severe anemia or other adverse events. All the patients in the study were 100% adherent to DAA treatment, as assessed using the Morisky-Green test.

### Psychiatric characteristics

At the start of DAA therapy, 46 (31.7%) patients had a previous history of psychiatric disease, as reported in the patient treatment form (S1 Annex). A further 16 (11%) patients had a current severe mental disorder according to WHO international classification of mental and behavioural disorders[25] (5 schizophrenia, 1 schizoaffective disorder, 3 major depressive disorder, 1 bipolar disorder, 2 adaptive disorder, 1 obsessive-compulsive disorder, 1 somatoform disorder, and 2 generalized anxiety disorder), as evaluated by the psychiatrist. At the start of DAA therapy, 42 (29.4%) patients were receiving psychiatric medication (antidepressants [*n* = 20; 54.1%], antipsychotics [*n =* 7; 18.9%], benzodiazepines [*n* = 10; 27%], or others [*n* = 5; 13.5%]). Twenty (16.3%) patients reported they had consumed >120g of alcohol per day for > 5 years. None of them were actively consuming alcohol at the start of treatment (Table 1).

### Laboratory test results and treatment adherence

Table 2 summarizes laboratory findings and treatment adherence over time (baseline, week 4, week 12, end of treatment, and 12 weeks after the end of treatment).

**Table 2:** Laboratory test results and treatment adherence.

Variable	Baseline	Week 4	Week 12	End of treatment	12 weeks after the end of treatment
Hemoglobin g/L (mean ± *SD)	140.76 ± 19.79	128.61 ± 22.54	127.33 ±17.97	126.98 ± 21.58	141.23 ± 19.72
Platelets ×10⁹/L (mean ± *SD)	146.15 ± 69.59	163.47 ± 77.16	136.92 ± 71.82	162.95 ± 75.21	156.78 ± 70.27
Viral load (RNA Log10 IU/ml) (mean ± *SD)	6.05 ± 0.75	1.37 ± 0.29	Undetectable	Undetectable	Undetectable
Albumin g/L (mean ± *SD)	42.09 ± 5.41	42.21 ± 4.81	41.46 ± 4.94	41.91 ± 5.18	43.68 ± 4.09
**INR (mean ± *SD)	1.25 ± 0.42	1.23 ± 0.35	1.41 ± 0.54	1.29 ± 0.52	1.21 ± 0.28
Bilirubin mg/dL (median****(IQR))	0.7 (0.7)	0.9 (0.9)	0.85 (1.05)	0.7 (0.9)	0.6 (0.45)
***ALT U/L (median****(IQR))	61 (55)	19 (10)	18 (11)	16 (9)	17.5 (10)
Neutrophils ×10⁹/L (mean ± *SD)	3.71(4.4)	3.58 (1.6)	3.36 (1.58)	3.48(1.68)	3.58(1.6)
Adherence	-	100%	100%	100%	-

*SD, Standard deviation

**INR, International Normalized Ratio

***ALT, Alanine aminotransferase

****IQR (interquartile range)

#### HADS evolution

**Fig 3:**
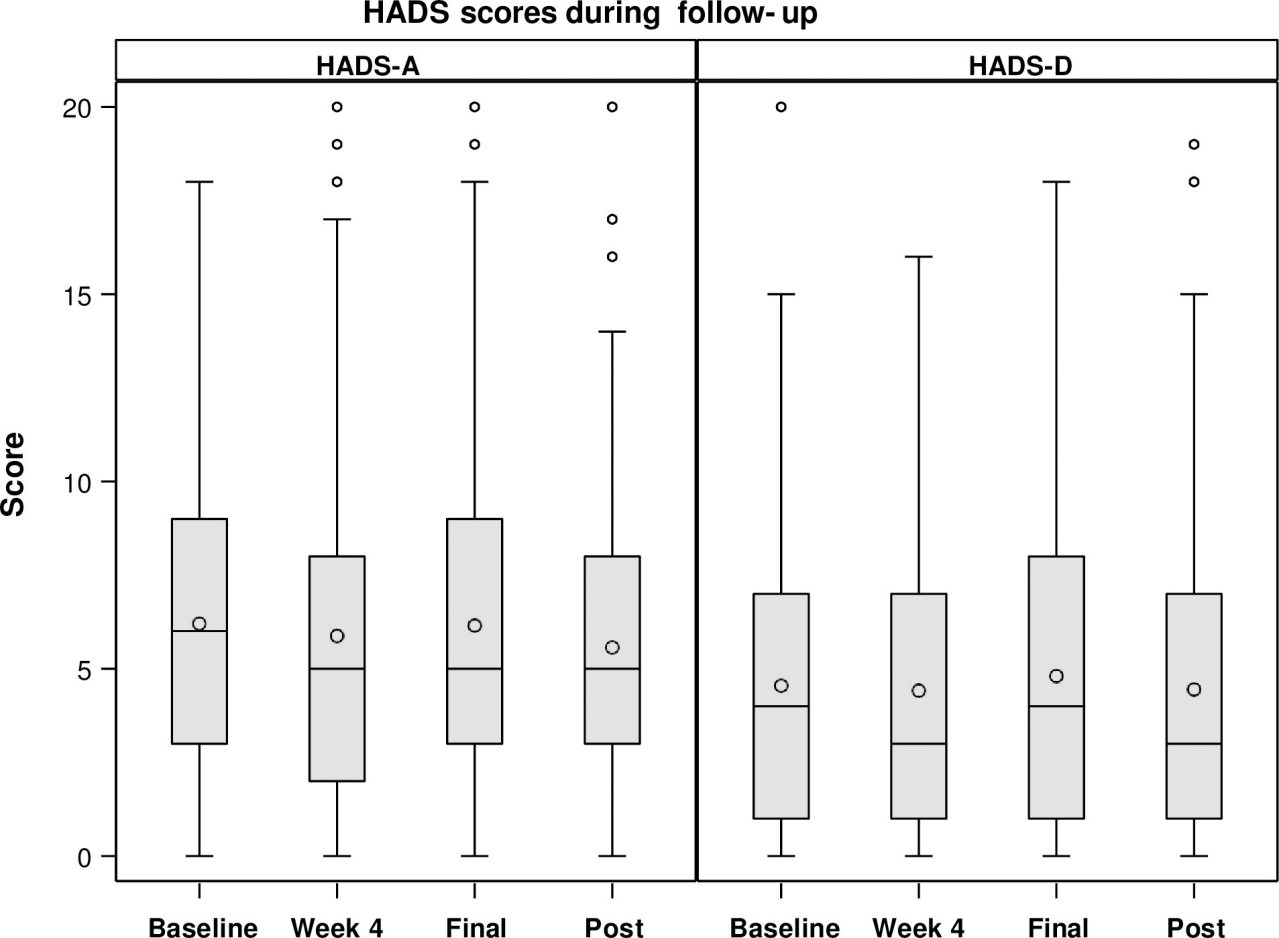
HADS scores during follow-up.

HADS-A values did not change significantly during follow-up (p = 0.19 and p = 0.62, respectively) (Fig 3). Values were: (baseline 6.20 ± 4.15; week 4, 5.88 ± 4.40; end of treatment, 6.14 ± 4.51; and 12 weeks after finishing treatment 5.57 ± 4.34) and HADS-D scores (baseline 4.55 ± 4.13; week 4, 4.42 ± 4.12; end of treatment, 4.80 ± 4.46; and 12 weeks after finishing treatment 4.45 ± 4.59). The results of patients who scored more or less or equal than 7 are reflected in Table 3.

**Table 3:** Results of patients with scores less than or equal to seven, or higher than seven.

	*HADS-A	*HADS-A	**HADS-D	**HADS-D
Variable (Row Pct)	≤ 7	> 7	≤ 7	>7
**Baseline** **N (%)**	93 (68.06)	44 (31.94)	104 (77.08)	33 (22.92)
**Week 4** **N (%)**	97 (68.06)	43 (31.94)	105 (73.61)	39 (26.39)
**End of treatment** **N (%)**	98 (68.28)	46 (31.72)	105 (74.48)	39 (25.52)
**Week 12 after the end of treatment** **N (%)**	51 (68.31)	21 (31.69)	58 (73.24)	16 (26.76)

*HADS-A, Hospital Anxiety and Depression Scale-Anxiety score

**HADS-D, Hospital Anxiety and Depression Scale-Depression score

Results of HADS-A and HADS-D did not change when different patient subgroups were analysed. Thus, when considering treatment duration (12 *vs*. 24 weeks), scores did not change significantly over time (*P* = 0.55and *P* = 0.88 for HAD-A and HAD-D, respectively). Moreover, no differences were detected between patients with significant fibrosis or cirrhosis versus absence of or mild fibrosis either (*P* = 0.80 and *P* = 0.64 for HADS-A and HADS-D, respectively) or when considering sex and age (*P* = 0.20 and *P* = 0.62 for HADS-A and HADS-D, respectively). The change in HADS scores was also analysed depending on ribavirin intake. No significant differences were reported in this regard (*P* = 0.18) (Table 4). Only one patient of those on ribavirin treatment had anemia at week 4 of treatment.

### HADS evolution in psychiatric patients

**Fig 4:**
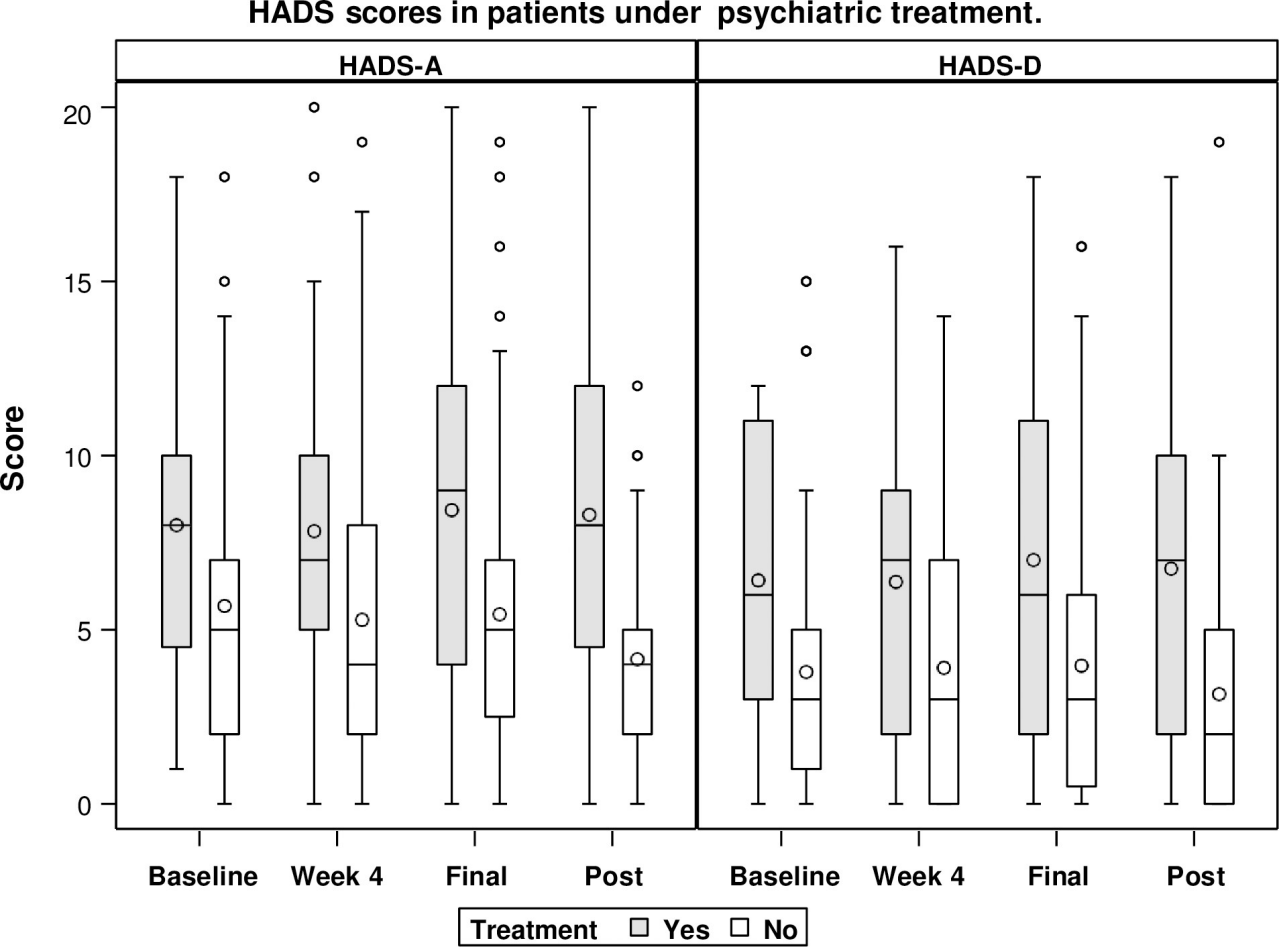
HADS scores in patients on psychiatric treatment. **HADS-A**, Hospital Anxiety and Depression Scale-Anxiety score; **HADS-D**, Hospital Anxiety and Depression Scale-Depression score.

In patients with a history of psychiatric disorders (*n* = 37), HADS-A scores were: baseline, 8 ± 3.99; week 4, 7.83 ± 4.50; end of treatment, 8.43 ± 4.92; and 12 weeks after finishing treatment, 8.30 ± 5.33; *P* = 0.92. HADS-D scores were: baseline, 6.42 ± 4.25; week 4, 6.37 ± 4.29; end of treatment, 7 ± 4.94; and 12 weeks after finishing treatment, 6.75 ± 5.23; *P* = 0.89). Moreover, scores in the subgroup of patients on psychiatric treatment (*n* = 42) did not change significantly during follow-up (*P* = 0.93 and 0.68, respectively) (Fig 4). The HAD did not change in patients with severe mental disorders (n = 16) during follow-up. HADS-A scores were: baseline, 7.8 ± 4.3; week 4, 6.8 ± 4.9; end of treatment, 7.1± 4.2; and 12 weeks after finishing treatment, 6.6 ± 4.0; P = 0.92. HADS-D scores were: baseline, 7.9 ± 5.1; week 5.4 ± 4.2; end of treatment, 6.3 ± 4.8; and 12 weeks after finishing treatment, 6.6 ± 4; P = 0.89)

**Table 4:** Change in Hospital Anxiety and Depression Scale scores according to ribavirin treatment.

Time treatment	*n*	Ribavirin	**HADS-A (Mean ± *SD)	***HADS-D (Mean ± *SD)
Baseline	82	Yes	6.18 ± 4.27	4.44 ± 4.10
	55	No	6.24 ± 4.00	4.71 ± 4.21
Week 4	80	Yes	6.10 ± 4.67	4.49 ± 4.03
	60	No	5.58 ± 4.02	4.32 ± 4.27
End of treatment	84	Yes	6.18 ± 4.95	5.15 ± 4.64
	60	No	6.08 ± 3.84	4.30 ± 4.17
12 weeks after the end of treatment	34	Yes	5.60 ± 5.16	4.66 ± 5.35
	38	No	5.54 ± 3.51	4.26 ± 3.85

*SD Standard deviation

** HADS-A Hospital Anxiety and Depresion Scale -Anxiety score

***HADS-D Hospital Anxiety and Depresion Scale- Depression score

### Efficacy of DAA treatment

The intention-to-treat analysis found an SVR in 140 (97.2%) patients; 5 (2.8%) patients relapsed after treatment. The SVR was 100% in both the subgroup of patients with a history of psychiatric disorders (*n* = 46) and the subgroup with current severe mental disorder (*n* = 16).

## Discussion

The present study confirms that DAA therapy does not affect anxiety or depression scores either during treatment or after achieving SVR, even in patients with a history of psychiatric disorder or current severe mental disorder. Some recent clinical series have reported improved quality of life in patients treated with DAAs, but these studies did not specifically measure anxiety or depression.[16, 32–34] Our results are new and in line with a more recent study that reported successful DAA treatment in psychiatric patients without increasing depressive symptoms.[15] The limitation of this study was the small sample size.

Another interesting finding in our study was the lack of changes in anxiety and depression scores in the patients on previous psychiatric treatment during DAA treatment. Before DAAs became available, these patients were unable to receive treatment for chronic HCV infection because of the neuropsychiatric adverse effects associated with pegylated interferon. In our study, one-third of our patients had a psychiatric disorder at the start of treatment, and many had been diagnosed with a severe psychiatric disorder and were on psychiatric medication. This high prevalence is in agreement with previous studies showing a higher percentage of psychiatric diseases in patients with chronic HCV infections.[18, 35, 36]Few studies have specifically reported their experience with DAAs and mental disease. In a systematic review of 11 studies before the introduction of DAAs, Sublette et al.[37] showed that patients with psychiatric disorders had comparable SVR rates to controls if they continued psychological therapy (mean 42%), although discontinuation rates for antiviral agents were high (14%– 48%). In a recent retrospective cohort study of 43 patients from five clinical trials who were diagnosed with mental health disease requiring antidepressants, antipsychotics, mood stabilizers, or other psychotropics and were treated with DAAs, the authors found not only a high SVR, but also an improvement in the Beck Depression Inventory score. Moreover, adherence was similar in psychiatric and non-psychiatric patients.[3] Our study corroborates these findings, with no worsening of anxiety or depression in patients with psychiatric disorders, and low treatment discontinuation rate.

Psychiatric symptoms are influenced by sex and age in many cases.[38, 39] In the present study, no significant differences were found in the HADS score when variables were analysed according to these parameters.

Advanced fibrosis or cirrhosis affects PROs, especially in patients with ascites, encephalopathy, and malnutrition,[40] and mental health is one of the variables measured in PRO questionnaires[5, 8]. We did not find changes in depression or anxiety during DAA treatment in this patient subgroup either, confirming psychiatric safety also in this high-risk population.

Our study also showed that the use of ribavirin (58% of our patients) did not play a role in anxiety or depression during the follow-up period. During the inclusion period of our study, ribavirin retained an important role in the optimal treatment of some patient subgroups, particularly those who have historically been considered more difficult to cure, such as genotype 3, decompensated cirrhosis or failure of previous treatment.[41] Ribavirin is known to cause anemia and other side effects that could impair quality of life, but the frequency and severity of these effects are considerably reduced and easy to manage when co-administered with DAAs in the absence of interferon[41]. Despite this, anemia can affect patients with psychiatric disorders,[42] and can cause cognitive function disorders and depression or exacerbate an existing psychiatric condition when left untreated.[43] Our study showed that the addition of ribavirin to DAA regimens had no effects on anxiety and/or depression during the treatment.

Our study has some limitations. We cannot rule out a potential bias due to physicians’ criteria for prescription, or to missing data resulting from incomplete patient records or data entry errors. Another limitation of our study is the lack of measure of quality of life or PRO. When the protocol was designed, we considered that additional questionnaires would represent an extra difficulty for patients. Consequently, and considering that our primary endpoint was the assessment of anxiety and depression, no additional questionnaires were included in the study. Another limitation is that, although our study suggests that a multidisciplinary team allows excellent adherence and cure rates to be obtained, this may not be generalizable to areas with fewer resources. Nursing care was very important in our experience to enhance patients’ knowledge and adherence. Furthermore, pharmacist monitoring of adherence is important for such expensive drugs. Finally, psychiatric evaluation of all patients with pathological HADS at the start of treatment could have affected the final HADS results by improving patient management. However, results of the study modified clinical practice in our unit, in that we were confident in reducing the intensity of psychiatric monitoring during treatment, limiting intervention to patients with newly detected or unstable psychiatric diseases. This study, therefore, reassures clinicians of the safety of DAAs, even in the special high-risk population.

In conclusion, our study shows that in clinical practice, DAA treatment had no impact on anxiety or depression in chronic HCV patients, even if they had previous or current psychiatric disorders or advanced fibrosis.

## Supporting information

S1 Annex Standardized form used to evaluate patients in the hepatology outpatient unit. (DOCX)
