# Correction: Impact of treatment with direct-acting antivirals on anxiety and depression in chronic hepatitis C

**DOI:** 10.1371/journal.pone.0241559

**Published:** 2020-10-26

**Authors:** Marta Gallach, Mercedes Vergara, Joao Pedro da Costa, Mireia Miquel, Meritxell Casas, Jordi Sanchez-Delgado, Blai Dalmau, Núria Rudi, Isabel Parra, Teresa Monllor, Meritxell Sanchez-Lloansí, Angelina Dosal, Oliver Valero, Xavier Calvet

An additional affiliation is missing for the 1st, 2nd, and 14th authors. Marta Gallach, Mercedes Vergara, and Xavier Calvet are affiliated with Departament de Medicina de la Universitat Autònoma de Barcelona, Barcelona, Spain.
