# Supplementary material for: Impact of treatment with direct-acting antivirals on anxiety and depression in chronic hepatitis C
Source: PLoS One. 2018 Dec 19;13(12):e0208112. doi: 10.1371/journal.pone.0208112 (PMC6300319; doi:10.1371/journal.pone.0208112)
Supplement: S1 Annex — (DOCX) [file pone.0208112.s001.docx]

**S1 Annex**

**Patient form for treatment**:

Sociodemographic data:

Name: ___________________________________________________________

Age: ________

Sex: Male Female

Marital status: Single Married/Living with partner Separated/Divorced

Number of children: __________

Number of persons with whom you live: ______________________

Place of birth: _________________

Highest educational level completed:
None Primary Secondary Tertiary

Employment status at the beginning of treatment:
Active UnemployedRetired Student Homemaker

Personal psychiatric history:

- Have you ever been on psychiatric or psychological treatment? ___________
             _______________________________________________________________

- When? _______________________________________________________

- Do you remember the medication you took? ______________________

- Are you under treatment today? __________________________________

- Which? __________________________________________________________
             
- What was your diagnosis? ____________________________________

- Have you ever tried to take your own life? ______________________________

- When? _______________________________________________________

- In what way? __________________________________________________

- Have you ever been hospitalized in a Psychiatric Unit? ___________________

- Have you ever abused alcohol for a prolonged period? _______________________________________________

- Have you ever used other drugs? _______________________________

Family psychiatric history:

- Has anyone in your family had a mental illness? Who? Do you know what illness? ______________________________________________________ _________

- Did they receive medication? Do you know what medication they received? __________________

- Has anyone in your family ever tried to take their own life? _____________
